# Barriers to Seeking Help for Skin Cancer Detection in Rural Australia

**DOI:** 10.3390/jcm6020019

**Published:** 2017-02-13

**Authors:** Kate M. Fennell, Kimberley Martin, Carlene J. Wilson, Camilla Trenerry, Greg Sharplin, James Dollman

**Affiliations:** 1 Flinders Centre for Innovation in Cancer, School of Medicine, Flinders University, Sturt Road, Bedford Park 5042, South Australia, Australia; carlene.wilson@flinders.edu.au (C.J.W.); camilla.trenerry@flinders.edu.au (C.T.); 2 Cancer Council SA, 202 Greenhill Road, Eastwood 5063, South Australia, Australia; kimberley.martin@sahmri.com (K.M.); greg.sharplin@unisa.edu.au (G.S.); 3 Freemasons Foundation Centre for Men’s Health, The University of Adelaide, Adelaide 5005, South Australia, Australia; james.dollman@unisa.edu.au; 4 Sansom Institute for Health Research, University of South Australia, Adelaide 5001, South Australia, Australia; 5 South Australian Health and Medical Research Institute, Adelaide 5001, South Australia, Australia; 6 School of Psychology, The University of Adelaide, Adelaide 5005, South Australia, Australia; 7 Alliance for Research in Exercise, Nutrition and Activity, School of Health Sciences, University of South Australia, Adelaide 5001, South Australia, Australia

**Keywords:** rural, barrier, help, help-seeking, skin, cancer, psychosocial, physician-patient relations

## Abstract

This study explores rural South Australians’ barriers to help-seeking for skin cancer detection. A total of 201 randomly selected rural adults (18–94 years, 66% female) were presented with a skin-cancer-related scenario via telephone and were asked the extent to which various barriers would impede their help-seeking, based on an amended version of the Barriers to Help-Seeking Scale. Older (≥63 years) and less educated participants endorsed barriers more strongly than their younger, more educated counterparts in the following domains; “Concrete barriers and distrust of caregivers”, “Emotional control”, “Minimising problem and Normalisation”, “Need for control and self-reliance” (every domain other than “Privacy”). Socioeconomic disadvantage, gender, and farmer status did not predict stronger overall barriers, but some gender and occupation-related differences were detected at the item level. Farmers were also more likely to endorse the “Minimising problem and normalization” domain than their non-farmer working rural counterparts. Widely endorsed barriers included the tendency to minimise the problem, a desire to remain in control/not be influenced by others, reluctance to show emotion or complain, and having concerns about privacy or waiting times.

## 1. Introduction

Attitudinal and contextual barriers are psychosocial factors that can prevent or delay people from seeking medical advice from physicians. Understanding these barriers from the perspective of potential patients may assist physicians to communicate in ways that promote future help-seeking. This paper explores attitudinal and contextual barriers to help-seeking from a general practitioner (GP) or other health professional, for the purpose of skin cancer detection, as an example.

In Australia, 80% of newly diagnosed cancers are either melanoma or non-melanoma skin cancers (i.e., basal cell carcinoma [BCC] or squamous cell carcinoma [SCC]), which is more than five times the global rate [1]. Melanoma can be fatal and, although non-melanoma cancers are often non-fatal, the latter can metastasise and cause considerable tissue damage [2], lead to disfigurement [3], decrease quality of life [4], and be very costly to the health system [1]. Therefore, the value of at-risk individuals having regular skin checks and seeking help early in response to changes to their skin cannot be overstated. 

A demographic group at particular risk of morbidity from skin cancer are those living away from capital cities in inner regional, outer regional, remote, and very remote areas of Australia [5]. More specifically, the incidence of non-melanoma lip cancer in rural Australia is approximately double that in urban populations, and invasive melanomas tend to be thicker when diagnosed in this population [6]. There is also a higher incidence of melanoma among rural females compared to urban females, and rural men with melanoma experience poorer outcomes than their male urban counterparts [1]. The cause of this inequality is currently not well understood. It may be due to the rural population’s work-related excess sun exposure (due to the nature of industries that tend to be located in rural areas) and the sun’s causal role in the development of BCC, SCC, and melanomas [1,7,8], or because people in rural areas tend not to check their skin often enough or delay help-seeking when they notice changes to their skin. 

Although it is widely acknowledged that rural populations, particularly farmers, have unique beliefs and attitudes that affect help-seeking patterns [9,10], to date, the demographic predictors and structural and attitudinal factors that impact the likelihood of seeking help for skin cancer related issues are poorly understood. Previous research has only focused on help-seeking for skin cancer in urban populations [11], overseas regional populations [12,13], overseas rural populations [10], and among rural Australians affected by cancer other than that of the lip or skin [14,15]. The majority of Australian research on rural residents’ barriers to health service support seeking has focused on barriers to mental health service use [16,17,18]. Therefore, the present study addresses a significant gap in the literature by identifying groups within the rural population who endorse barriers most strongly and by determining the ten most frequently endorsed barriers to help-seeking in this context. It also seeks to identify the specific barriers (and both the domain and item levels) that are most problematic within these groups and thereby lay the foundation for the development of strategies for use by physicians and public health professionals to increase early help-seeking and early intervention. Given that skin cancers are often “uniquely susceptible to prompt action and cure” [19] (p. 591), early detection can deliver better health outcomes [19] and because rural Australians are clearly a unique [9,10] and at-risk group [1,6], this is an important area of enquiry. 

## 2. Methods

### 2.1. Participants

In 2014, adults (≥18 years) from three rural regions in South Australia (the Riverland, Copper Coast/Yorke Peninsula, and Eyre Peninsula) were randomly selected from the Electronic White Pages and invited to participate in a Computer Assisted Telephone Interview (CATI). Up to five attempts to contact potential participants were made before a non-response was recorded (14% of all calls). The final sample included 201 participants, with a participation rate of 40%. There were no exclusion criteria. 

### 2.2. Instruments

#### 2.2.1. Barriers to Help-Seeking

The Barriers to Help Seeking Scale (BHSS) is a 31-item measure that was originally designed in the United States to assess the reasons men do not seek help for physical and mental health problems. Previous research has demonstrated that it is reliable, has good internal consistency, as well as good convergent and criterion validity when compared with other measures of help-seeking [20].

The following domains were represented in the adapted version of the BHSS [20] used in this study: Need for Control and Self-Reliance (12 items, Cronbach’s alpha (α) = 0.88); Concrete Barriers and Distrust of Caregivers (12 items, α = 0.81); Minimising Problem and Normalisation (7 items, α = 0.73); Privacy (4 items, α = 0.65); and Emotional Control (4 items, α = 0.76). Items 23, 24 (Need for Control and Self-Reliance domain), and 31 (Minimising Problem and Normalisation domain) were added to the original BHSS items as our literature review identified them as commonly cited barriers to help seeking in rural Australia, but they were not covered in the original measure. 

#### 2.2.2. Demographic Information

Age, gender, postcode of residence to determine Index of Relative Socio-economic Disadvantage (IRSD) [21] which we refer to as “SED” (in other contexts this may be known as socio-economic status), highest level of educational attainment (never attended; some primary school; completed primary school; some high school; completed Year 12 at high school; trade or diploma; university or other tertiary qualification), work status (self-employed; employed for salary or wage; unemployed; home duties; student; retired; unable to work) and occupation were collected at the start of the CATI.

### 2.3. CATI Procedure

Upon commencement of the interview, appropriate study information was provided to participants and verbal consent was sought. Subsequent to this, participants were asked to respond to a series of demographic questions and were then randomly allocated to one of two skin cancer related scenarios. The scenarios were adapted from the original bodily-pain scenario presented in the BHSS [20]. 

*Scenario 1: Imagine that you notice a new spot on your skin or an old spot that has changed in colour, shape or size*.

*Scenario 2: Imagine that you have not noticed any obvious changes to your skin but know that it has been a while since you had your skin checked for skin cancers*.

The interviewer then continued with the following script: *You consider seeking help from a medical doctor or other healthcare provider. I am going to read out several reasons why you might choose not to seek help. Please consider each reason and decide how important it would be in keeping you from seeking help.* This script was repeated midway through the interview to remind them of the response context. Participants were instructed to respond to each reason for choosing not to seek help (from the adapted BHSS) with; “strongly disagree”, “disagree”, “neither agree nor disagree”, “agree”, or “strongly agree” and they were recorded on a five-point scale from 1 “strongly disagree” to 5 “strongly agree”.

All data were collected between 17th and 29th September 2014. Ethics approval was granted by The University of South Australia’s Human Research Ethics Committee (Application ID: 0000033418).

### 2.4. Statistical Analyses

The majority of data analyses were undertaken using SPSS version 20, with results considered significant at the *p* < 0.05 level. Participants were divided into older (63 years and over) and younger age groups based on a median split. Similarly, participants were divided into “more disadvantaged” (IRSD Quintiles 1 and 2) and “less disadvantaged” (IRSD Quintiles 3 and 4) groups. The quintile of least disadvantage (IRSD Quintile 5) was excluded because no respondents resided in an area classified in this quintile.

Domain scores were calculated by averaging individual item responses. Averages were employed rather than raw totals so that comparisons between domains with different numbers of items could be compared. Higher scores represented a stronger endorsement that an item (or a domain of items) was an important barrier to help-seeking. An overall/total barriers score was calculated as the average of the domain scores so that straightforward comparisons could be made between demographic groups about the extent to which they endorsed barriers overall. 

Preliminary analyses revealed only minor differences in the responses of the group that received Scenario 1, compared to the group that received Scenario 2 (scores differed significantly by scenario for only 2 of the 39 barrier items). Therefore, responses to the two scenarios were combined for all subsequent analyses. 

*t*-Tests were used to determine differences in the means of the overall/total barrier and domain scores by demographic characteristics. The same *t*-test analyses were also performed on individual items comprising the domains to enable exploration of more specific barriers/issues that may have been lost at the domain level. While overall barrier and domain level analyses are deemed important to provide foundations for future comparisons with other samples (e.g., internationally), the finer grained, item by item analysis allows us to highlight specific, actionable interventions that are likely to be helpful and translatable for policy makers and health professionals. All demographic variables were also entered into a simultaneous multiple regression for each domain to examine the predictive contribution of each demographic factor to variance in domain scores. 

As domain scores provide no absolute scale of barrier strength, the percentages of respondents who agreed by indicating that they “agreed” (4) or “strongly agreed” (5), with the barrier represented by each *item* were calculated and presented descriptively. 

## 3. Results

### 3.1. Demographic Characteristics of the Sample

The sample had a mean age of 61.7 years (*SD* = 15.4) and median age of 63 years, ranging from 18 to 94 years. Table 1 displays the demographic characteristics of the sample (*N* = 201). We compared the characteristics of our sample with those of the broader Australian population using representative population-based data collected by the Australian Bureau of Statistics [22,23]. As is often the case in health research [24], females were over-represented in our sample. Our sample was also older than the general Australian population and the population of rural South Australia [25]. We believe this can be simply explained by our sampling method and younger people’s preference for mobile phones [26], which are rarely included in telephone directories. Furthermore, people with high school, trade, and/or tertiary qualifications and people who currently worked, were underrepresented which may be explained by our sample’s greater average age. 

Total mean barrier domain scores and barrier domain scores by demographic group are displayed in Table 2.

### 3.2. Socioeconomic Disadvantage, Education, and Domain Scores

As displayed in Table 2, there were no significant differences in overall barrier scores or any of the domain scores between those of higher and lower socioeconomic disadvantage. Analysis at the individual item level also confirmed the equivalency of these groups. However, participants with lower levels of education had higher mean scores (i.e., endorsed barriers more strongly overall) than those with higher levels of education in the domains; “Concrete barriers and distrust of caregivers” (*t*(199) = 2.73 *p* = 0.007), “Emotional control” (*t*(199) = 2.54 *p* = 0.012), “Minimising problem and normalization” (*t*(199) = 2.97 *p* = 0.003), and “Need for control and self-reliance” (*t*(199) = 3.14 *p* = 0.002). Those with lower education also reported higher total barriers scores (*t*(199) = 3.13 *p* = 0.002) and the differences were also scattered across domains at the item level, in all but the “Privacy” domain (Table 3).

### 3.3. Gender and Domain Scores

There were no significant differences in mean scores across any of the domains by gender but as displayed in Table 3, differences did emerge at an item level.

### 3.4. Farmer Status and Domain Scores

There were no significant differences in total barrier scores between farmers and non-farmers in paid employment (*n* = 94). However, farmers had a significantly higher mean score for the “Minimising problem and normalization” domain compared to non-farmers (*t*(32.84) = −2.95 *p* = 0.006) and, at an item level, as displayed in Table 3, some significant differences between groups were also evident.

### 3.5. Age Group and Domain Scores

Participants aged 63 years and older endorsed barriers (total barrier scores) significantly more strongly than their younger counterparts (*t*(198) = −3.31 *p* = 0.001), as well as endorsing the following domains more strongly; “Concrete barriers and distrust of caregivers” (*t*(198) = −2.00 *p* = 0.047) “Emotional control” (*t*(198) = −3.07 *p* = 0.002), “Minimising problem and normalization” (*t*(198) = −2.44 *p* = 0.016), “Need for control and self-reliance” (*t*(198) = −4.81 *p* < 0.001). At an item level, several significant differences in barrier endorsement between age groups were also identified (see Table 3). 

### 3.6. Predictive Effect of Demographic Variables on Barrier Domain Scores

The combined effect of the demographic variables (outlined in Table 2) significantly contributed to variance in mean scores for all barrier domains except “Privacy”: “Concrete barriers and distrust of caregivers” (*R*^2^ = 0.088, *F*(6, 188) = 3.04, *p* = 0.007), “Emotional control” (*R*^2^ = 0.088, *F*(6, 188) = 3.03, *p* = 0.008), “Minimising problem and normalization” (*R*^2^ = 0.090, *F*(6, 188) = 3.08, *p* = 0.007), “Need for control and self-reliance” (*R*^2^ = 0.184, *F*(6, 188) = 7.06, *p* < 0.001) and total barrier score (*R*^2^ = 0.109, *F*(6, 188) = 3.82, *p* = 0.001). However, age (as a continuous variable) was the only demographic variable that was an independent significant predictor for variance in barrier domain scores. Age was a significant predictor in the “Concrete barriers and distrust of caregivers” (*R*^2^ = 0.22, *p* = 0.017), “Minimising problem and normalization” (*R*^2^ = 0.19, *p* = 0.038), and Need for control and self-reliance (*R*^2^ = 0.36, *p* < 0.001) domains and total barrier endorsement score (*R*^2^ = 0.23, *p* = 0.012).

### 3.7. Ten Strongest Barriers at the Item Level

The ten most strongly endorsed barriers to help-seeking for skin cancer detection in this population (at the item level) are detailed in Table 4. All of these were endorsed by at least 35% of the participants.

## 4. Discussion

This study yielded a mixture of expected and unexpected insights into barriers to help-seeking for skin cancer detection in the rural South Australian population. Although no directly comparable research exists, it was surprising to find that higher education significantly predicted lower total barrier scores in our rural sample *but* lower socioeconomic disadvantage did not. The finding that socioeconomic disadvantage did not predict barriers to help-seeking in our sample conflicts with Eiser et al.’s [11] finding in a UK sample (where, like in Australia, health care is heavily subsidized), that people who are more affluent are more willing to visit their general practitioner for a free skin check, than those from more disadvantaged groups. However, there was not a large variation of disadvantage in our sample as our measure was based on postcode, so using education as a proxy for socioeconomic disadvantage may have been more useful. The finding that lower education predicted higher barriers is consistent with Kannan and Veazie’s [27] research in the US; they found that less educated people were more likely to avoid healthcare when they need it. Similarly, in a sample of 513 Greeks, Kakagia et al. [28] found that lower education predicted delayed diagnosis of SCC.

The lack of differences between men and women on total barrier scores and at the domain level is surprising given the gender differences identified by previous research [11,27,28], but men did more strongly endorse some *individual* barriers than woman did. Furthermore, consistent with Emery et al.’s [14] findings that stoicism/machismo frequently prevented rural Western Australian males from seeking early help for symptoms of tumours (e.g., prostate and colorectal cancer), men scored significantly higher on some of the “Need for Control”-related items; “I don’t like other people telling me what to do” and “I like to make my own decisions and not be influenced by others” as well as items related to Self-Reliance; “I don’t like to get emotional about things” and “problems like this are just a part of life” (but they did not score higher on the overall domain). Targeting control and stoicism when communicating messages on the importance of skin-cancer detection to rural males may therefore be a useful strategy to test, as would be exploring stoicism and control in this population with more rigorous, validated measures (rather than single items which we were limited to in the present study). Males also scored higher on the item “I don’t know how to talk about something like this”, which suggests that educating rural men on how to have conversations about their skin cancer risk and possibly, more generally about other early detection/prevention issues, may be of use to them.

Given the results of meta-analyses suggesting that farmers are a subsection of the rural population who are at particular risk of developing skin [29] and lip [30] cancer, and even though we only had a very small sample, we were surprised to find that farmers did not report stronger overall barrier scores than non-farmers. This conflicts with the results of Judd et al. [31] who attribute the disproportionately high rate of suicide in the farming population to (at least in part), the farming community’s particularly negative attitudes towards help-seeking. However, it is possible that the farming community faces different barriers to help-seeking for preventative, physical health and mental health issues. This is worth investigating. It is also possible that our sample of farmers was too small to detect differences or that farmers’ greater risk of skin and lip cancer is not associated with barriers but instead explained by greater sun exposure. However, when examined at the domain level, farmers did report significantly higher scores in the “Minimise the Problem and Normalisation” domain, which is consistent with Judd et al.’s [31] finding that farmers tend to be less “neurotic” than their rural non-farming counterparts. This comparison would benefit from replication in a larger sample of farmers and non-farming rural workforce members.

As recent research by Kannan and Veazie [27] found that older Americans are *less* likely to avoid health care when they know they need it than younger Americans, our robust finding that those in older rural age groups report *stronger* barriers to seeking help for skin cancer detection was also surprising. However, Kannan and Veazie’s study [27] was not specific to rural people or skin cancer, highlighting the need to focus research on the barriers to help-seeking for specific conditions and in specific contexts. Consistent with our results, Eiser et al. [11] found that people over 50 years of age were more optimistic about avoiding skin cancer than those less than 50 years of age. Greater optimism about avoiding skin cancer is a variable worth exploring in the rural population. It is possible that younger rural South Australians are less optimistic about avoiding skin cancer due to their exposure to Australian SunSmart skin cancer awareness campaigns from a younger age [32].

Examination of the ten most widely endorsed items highlighted barriers associated with minimising the problem, a desire to remain in control/not be influenced by others, reluctance to show emotion or complain, stoicism and concerns about privacy and waiting times. These findings are consistent with the results of Emery et al.’s work [14] which concluded that among people just diagnosed with breast, lung, prostate, or colorectal cancer in rural Western Australia, stoicism, machismo, embarrassment, and fear all contributed to later presentations to their doctors with cancer-related symptoms. Examining and mapping the strength of social and attitudinal influences on help-seeking for skin cancer detection based on levels of isolation or remoteness would be useful given American evidence that the more isolated a rural community is, the more likely that its residents will hold stigmatised attitudes towards seeking mental health care [33], and the lack of research of this type in the Australian cancer prevention and early detection context.

Wide endorsement of the items “Problems like this are part of life (they’re just something you have to deal with)” and “I don’t like to get emotional about things” is also consistent with previous research that has shown that fatalistic attitudes [10,28,34] and stoic beliefs [10,14] act as barriers to help-seeking in rural populations. Belfort et al. [34] found in a large US sample that even when other demographic characteristics were controlled for, rural residents were significantly more likely to endorse fatalistic beliefs about cancer prevention than those in their urban sample. Similarly, Kakala et al. [28] found that fatalism predicted delayed presentation for SCC in a mixed rural/urban Greek sample. 

“It takes too long to get in and see my doctor/health professional” was the only structural barrier that was endorsed by more than 35% of the sample. This is consistent with Emery et al.’s [14] Australian qualitative research that highlighted health professional shortages and difficulty in making appointments as barriers to help-seeking in response to cancer symptoms in rural Western Australia. However, other practical and structural barriers such as “I can’t afford time off from work to seek help” and “I would have real difficulty finding transportation to a place where I can get help” were not widely endorsed by our sample. This finding conflicts with the work of Emery et al. [14] and Hall et al. [35], who found that transport/distance and time were important barriers in rural contexts. However, in Hall et al.’s [35] study it was rural GPs who cited distance as a barrier for rural patients seeking help for lung cancer, not the patients themselves. This may be explained by research from Corboy, McLaren, and McDonald [36] who concluded that health professionals tend to report barriers to help-seeking in rural areas as being structural or system-based, while rural patients are less concerned about issues such as travel and are more concerned with social factors and/or attitudinal barriers [36]. This highlights the need to explore barriers to engagement with health services from both the physicians’ and patients’ perspectives. Such barriers also need to be explored in different countries where health care systems work differently (e.g., cost may act as a barrier in the United States, but it is unsurprising that it is not highlighted in the present study given Australian’s universal access to health care). Privacy was also a strongly endorsed item level barrier to help-seeking in this Australian sample. Privacy has been raised as a concern in previous research involving people living in rural communities with other types of cancer [37,38,39,40], but to the best of our knowledge has not been previously identified as a barrier to help-seeking for skin cancer detection in this context. 

Some limitations of the present study should be noted. The BHSS was initially developed for use with males and utilised U.S. male college students in the scale validation. While every attempt was made to make the scale appropriate for use in the Australian population and internal consistency remained high on all but the privacy domain, being able to use a measure that has been designed specifically for the Australian population and has been validated in this context would be useful. With hindsight, the inclusion of some disease-specific barriers (e.g., over confidence in self-assessment, optimism that a mole is not a melanoma) such as those that Eiser et al. [11] included in their work, and the inclusion of measures of *facilitators* of help-seeking (e.g., possibly social support) would also have been useful additions. Other potential limitations are that participants were asked to respond based on a hypothetical scenario, rather than their lived experience; our “young” category was not particularly young; we had insufficient statistical power to assess interactions at multiples levels of demographic variables; and we did not collect data on actual health service use in the context of skin cancer. This is an issue because research generally shows that help-seeking intention is not a perfect predictor of help-seeking behavior (e.g., [18]). Furthermore, recruiting via the electronic white pages means there is likely to be selection bias towards households with functioning landlines. 

Nonetheless, this novel study provides insights into barriers that might be considered by physicians when communicating with their patients and that may be targeted at a public health level, via education campaigns. Importantly, it identifies the most dominant barriers for the rural population as a whole, and those that are particularly powerful in specific rural groups. The finding that the statement “It takes too long to get in and see my doctor/health professional” was endorsed by over 40% of the sample suggests the need for advocacy for structural changes to healthcare in rural South Australia, to help in decreasing the burden of skin and lip cancer in this at-risk population. Increasing the availability of rural GPs may also improve outcomes for other health conditions that benefit from early detection and/or can be prevented.

## 5. Conclusions

In summary, the results of this study suggest that rural Australians with lower levels of education and those who are 63 years of age and over, are more likely to strongly endorse barriers to help-seeking for skin cancer detection than their more educated and younger counterparts. Older and less educated individuals scored higher on “Need for control/self-reliance”, “Minimising the problem and normalization”, “Emotional control”, and “Concrete barriers and distrust of caregivers”. Socioeconomic disadvantage, gender, and farmer status were not associated with differences in overall assessment of barriers and only farmer status was associated with a significant difference in a domain score (i.e., “Minimising problem and normalization” was a stronger barrier for farmers than for other working participants). Age was found to be a particularly strong predictor of barrier endorsement at the domain level, with a clear link between older age and high levels of “Emotional control”, “Concrete barriers and distrust of caregivers”, “Minimising the problem and normalization”, and the “Need for control and self-reliance”. More generally, widely endorsed barriers included the tendency to minimise the problem, a desire to remain in control/not be influenced by others, reluctance to show emotion or complain, and having concerns about privacy or waiting times. Structural issues such as not being able to afford time away from work and difficulties with transport were among the most rarely endorsed barriers. 

Together these findings also highlight that rural populations are unique, and what is known about barriers to skin cancer detection in the broader population, and barriers to help-seeking for health and mental health conditions in the rural population more broadly, do not necessarily apply to rural people who are considering whether or not to talk to health professionals about their skin. While further research in this field with more detailed and valid instruments is required, this study demonstrates that the rural population is not homogenous and that efforts to promote skin cancer awareness and detection interventions are likely to be more effective if they address the barriers that are particularly dominant within certain demographics of this at-risk group.

## Figures and Tables

**Table 1:** Demographic characteristics of participants.

Demographic Categories	% ^†^ (*n* = 201)
**Age**	
18–34	3.5
35–54	28.5
55–74	48.0
75+	20.0
**Gender**	
Male	33.8
Female	66.2
**Highest level of education achieved**	
Completed primary school	5.5
Some high school	44.3
Completed high school	15.9
TAFE, trade certificate or diploma	21.9
University or other tertiary institute degree	12.4
**Work status**	
Not currently in paid employment	53.2
Retired	42.8
Home duties	5.5
Unable to work	3.0
Unemployed	1.5
Currently in paid employment	46.8
**Occupation** ^^^	
Farmer	16.0
In non-farming paid employment	84.0
**Quintile of disadvantage**	
1 Most disadvantaged	45.9
2	29.6
3	8.7
4	15.8
5 Least disadvantaged	0.0

† valid percentages reported; ^ amongst those in paid employment (*n* = 94).

**Table 2:** Mean domain barrier scores # (SDs), by demographic factors.

Domain	Age Group		Gender		Education		Socioeconomic Disadvantage		Work Status		Farmer Status ^†^		Total (*n* = 201)
	Under 63 (*n* = 96)	63 and over (*n* = 104)	Male (*n* = 68)	Female (*n* = 133)	Did not complete HS (*n* = 100)	Completed HS and above (*n* = 101)	More disadvantaged (*n* = 148)	Less disadvantaged (*n* = 48)	Not in paid employment (*n* = 107)	In paid employment (*n* = 94)	Non-farmer (*n* = 79)	Farmer (*n* = 15)	
**Concrete barriers and distrust of caregivers**	1.93 (0.57)	2.11 * (0.69)	2.08 (0.59)	1.99 (0.67)	2.15 ** (0.70)	1.90 (0.56)	2.05 (0.65)	1.97 (0.64)	2.11 * (0.69)	1.92 (0.57)	1.88 (0.56)	2.13 (0.61)	2.02 (0.64)
**Emotional control**	2.46 (0.92)	2.86 ** (0.91)	2.82 (0.95)	2.59 (0.92)	2.84 * (0.92)	2.50 (0.93)	2.64 (0.94)	2.72 (0.93)	2.82 * (0.94)	2.50 (0.91)	2.49 (0.92)	2.55 (0.88)	2.67 (0.94)
**Privacy**	2.29 (0.75)	2.32 (0.78)	2.25 (0.78)	2.34 (0.75)	2.34 (0.81)	2.27 (0.72)	2.29 (0.77)	2.35 (0.78)	2.36 (0.80)	2.25 (0.72)	2.27 (0.75)	2.16 (0.55)	2.31 (0.76)
**Minimising problem and normalisation**	2.30 (0.73)	2.54 * (0.64)	2.50 (0.60)	2.39 (0.74)	2.57 ** (0.69)	2.29 (0.67)	2.43 (0.68)	2.46 (0.75)	2.47 (0.70)	2.38 (0.69)	2.31 (0.71)	2.70 ** (0.41)	2.43 (0.69)
**Need for control and self-reliance**	2.05 (0.69)	2.55 *** (0.76)	2.47 (0.76)	2.24 (0.77)	2.49 ** (0.79)	2.48 ** (0.59)	2.31 (0.76)	2.35 (0.78)	2.48 ** (0.78)	2.13 (0.72)	2.09 (0.75)	2.34 (0.56)	2.32 (0.77)
**Total barriers**	2.21 (0.57)	2.47 ** (0.57)	2.42 (0.54)	2.31 (0.61)	2.15 (0.73)	2.22 (0.56)	2.34 (0.57)	2.37 (0.63)	2.45 * (0.58)	2.24 (0.58)	2.21 (0.60)	2.38 (0.43)	2.35 (0.59)

* *p* < 0.05 ** *p* < 0.01 *** *p* < 0.001; # “strongly disagree” = 1, “disagree” = 2, “neither agree nor disagree” = 3, “agree” = 4 or “strongly agree” = 5; ^†^ amongst those currently in paid employment; HS = High school (secondary education).

**Table 3:** Individual item barrier mean scores and variation in scores by demographic factors.

Q #	Item/Domain	Rank (1–39)	Mean	SD	Gender	Age Group	Education	Farmer Status ^†^
	**Concrete barriers and distrust of caregivers domain**						** less	
8	It takes too long to get in and see my doctor/health professional	7	2.87	1.45				
7	I prefer to get help from family and friends	19	2.20	1.29		** older	** less	
9	I find it difficult to understand my doctor/health professional	20	2.16	1.19				
3	Financial difficulties would be an obstacle to getting help	22	2.10	1.19				
11	I’m worried about the side effects of what they might do	23	2.06	1.13			* less	
5	I can’t afford to have time off from work to seek help	25	2.02	1.08				
2	I wouldn’t know what sort of help was available	26	2.01	1.16		*** older	*** less	
1	I would have real difficulty finding transportation to a place where I can get help	31	1.91	1.15		** older		* farmer
10	I prefer to get information or help from the internet or over the phone	33	1.78	0.92				
6	I don’t have someone to come to the appointment with me	34	1.77	0.98			* less	
4	I don’t trust doctors and other health professionals	35	1.74	0.94				
12	I don’t know how to talk about something like this	36	1.67	0.84	* male	* older	** less	
	**Emotional control domain**					** older	* less	
36	I don’t like to get emotional about things	6	2.97	1.26	* male	* older	* less	
38	I’d rather not show people what I’m feeling	8	2.85	1.23			* less	
37	I don’t like to talk about feelings	11	2.59	1.23		** older		
39	I wouldn’t want to look stupid for not knowing how to figure this problem out	17	2.27	1.22		* older		
	**Privacy domain**							
32	Privacy is important to me, and I don’t want other people to know about my problems	3	3.00	1.30				* farmer
34	I am worried about confidentiality in our community	16	2.28	1.13				
33	This problem is embarrassing	27	2.00	0.94				
35	I am worried that I might know the healthcare provider outside of their work	30	1.95	0.98				
	**Minimising problem and normalisation domain**					* older	** less	** farmer
31	Most people in the region have got at least one health issue they are dealing with	*1*	3.66	1.13				
28	Problems like this are part of life (they’re just something you have to deal with)	*2*	3.12	1.35	** male	*** older	* less	
27	I wouldn’t want to overreact to a problem that wasn’t serious	12	2.50	1.25			* less	
29	I’d prefer just to suck it up rather than dwell on my problems	18	2.23	1.16			** less	
30	I would prefer to wait until I’m sure the health problem is a serious one	24	2.05	1.20				
25	The problem wouldn’t seem worth getting help for	32	1.86	0.96				
26	The problem wouldn’t be a big deal (it would go away in time)	37	1.65	0.75				* farmer
	**Need for control and self-reliance domain**					*** older	** less	
20	I like to be in charge of everything in my life	4	3.00	1.31		*** older		
19	I like to make my own decisions and not be too influenced by others	5	2.99	1.33	* male	** older	** less	* farmer
23	I do not want to sound like I’m complaining	9	2.64	1.30				
17	I don’t like feeling controlled by other people	10	2.63	1.34		** older	* less	
24	I can still function so I don’t need help	13	2.48	1.25		** older	* less	
15	Nobody knows more about my problems than I do	14	2.47	1.31		** older		
16	I’d feel better about myself knowing I didn’t need help from others	15	2.32	1.20		*** older	** less	
14	I don’t like other people telling me what to do	21	2.10	1.16	*** male	*** older	** less	
22	I do not want to appear weaker than my peers	28	1.99	1.10		*** older	** less	
21	Asking for help is like surrendering authority or control over my life	29	1.96	1.07		* older		
18	It would seem weak to ask for help	38	1.65	0.75				
13	I would think less of myself for needing help	39	1.60	0.81		* older	* less	

* *p* < 0.05 ** *p* < 0.01 *** *p* < 0.001 denotes significantly higher barrier scores amongst demographic groups (“less” education refers to those that did not complete high school vs. completed high school, “older” refers to 63 years and over vs. under 63 years, “not paid” = not in paid employment vs. in paid employment). # “strongly disagree” = 1, “disagree” = 2, “neither agree nor disagree” = 3, “agree” = 4 or “strongly agree” = 5; ^†^ amongst those currently in paid employment; N/A = not applicable.

**Table 4:** Proportion (%) of sample that strongly agreed/agreed that the barrier was important and associated domain.

Item	*N* = 201% That Agree/Strongly Agree	Domain
Most people in the region have got at least one health issue they are dealing with	71.1	Minimising problem and normalisation
Problems like this are part of life (they’re just something you have to deal with)	57.7	Minimising problem and normalisation
I like to make my own decisions and not be too influenced by others	51.7	Need for control and self-reliance
I don’t like to get emotional about things	49.3	Emotional control
I like to be in charge of everything in my life	48.8	Need for control and self-reliance domain
Privacy is important to me, and I don’t want other people to know about my problems	47.8	Privacy
I’d rather not show people what I’m feeling	43.3	Emotional control
It takes too long to get in and see my doctor/health professional	41.8	Concrete barriers and distrust of caregivers
I do not want to sound like I’m complaining	35.8	Need for control and self-reliance
I don’t like feeling controlled by other people	35.3	Need for control and self-reliance
